# A Gold Growth-Based Plasmonic ELISA for the Sensitive Detection of Fumonisin B_1_ in Maize

**DOI:** 10.3390/toxins11060323

**Published:** 2019-06-05

**Authors:** Shengnan Zhan, Lingyan Zheng, Yaofeng Zhou, Kesheng Wu, Hong Duan, Xiaolin Huang, Yonghua Xiong

**Affiliations:** 1 State Key Laboratory of Food Science and Technology, Nanchang University, Nanchang 330047, China; zsn980078915@163.com (S.Z.); zhenglingyan0593@163.com (L.Z.); ncuskzhouyaofeng@163.com (Y.Z.); duanhong202@163.com (H.D.); hxl19880503@163.com (X.H.); 2 Jiangxi Institute of Veterinary Drug and Feedstuff Control, Nanchang 330096, China; 3 Jiangxi-OAI Joint Research Institute, Nanchang University, Nanchang 330047, China

**Keywords:** plasmonic enzyme-linked immunosorbent assay, controlled growth kinetics, glucose oxidase, naked-eye detection, fumonisins B_1_

## Abstract

In this paper, a highly sensitive plasmonic enzyme-linked immunosorbent assay (pELISA) was developed for the naked-eye detection of fumonisin B_1_ (FB_1_). Glucose oxidase (GOx) was used as an alternative to horseradish peroxidase as the carrier of the competing antigen. GOx catalyzed the oxidation of glucose to produce hydrogen peroxide, which acted as a reducing agent to reduce Au^3+^ to Au on the surface of gold seeds (5 nm), This reaction led to a color change in the solution from colorless to purple, which was observable to the naked eye. Various parameters that could influence the detection performance of pELISA were investigated. The developed method exhibited a considerably high sensitivity for FB_1_ qualitative naked-eye detection, with a visible cut-off limit of 1.25 ng/mL. Moreover, the proposed pELISA showed a good linear range of 0.31–10 ng/mL with a half maximal inhibitory concentration (IC_50_) of 1.86 ng/mL, which was approximately 13-fold lower than that of a horseradish peroxidase- (HRP)-based conventional ELISA. Meanwhile, the proposed method was highly specific and accurate. In summary, the new pELISA exhibited acceptable accuracy and precision for sensitive naked-eye detection of FB_1_ in maize samples and can be applied for the detection of other chemical contaminants.

## 1. Introduction

Fumonisins (FBs), a group of mycotoxins that are mainly produced by a number of *Fusarium* species, occur worldwide in foods, such as maize and maize-based products. Their presence in food leads to cases of acute and chronic exposure in humans and animals [1]. To date, 28 fumonisins have been isolated, and they can be categorized into four series, known as A, B, C, and P. Fumonisin B_1_ (FB_1_), B_2_ (FB_2_), and B_3_ (FB_3_) are the principal fumonisins that are the relevant contaminants of cereal [1,2]. Among them, FB_1_ has already been classified as a potential carcinogen (group 2B) by the International Agency for Research on Cancer because of its carcinogenic, nephrotoxic, and hepatotoxic effects on humans and animals [3]. The European Commission has set action levels for a sum of 4000 μg/kg FB_1_ in unprocessed maize, and maximum levels at 200 μg/kg in processed maize-based and baby foods [4,5]. A maximum level of 2.0–4.0 mg/kg for total fumonisins (FB_1_, FB_2_, and FB_3_) in human foods is recommended by the U.S. Food and Drug Administration [5,6].

Various instrumental detection methods, including the high performance liquid chromatographic method [4,7] and liquid chromatography coupled to mass spectrometry, or tandem mass spectrometry (LC/MS/MS) [8,9], have been developed for FB_1_ detection in different food samples. These methods have high accuracy, good specificity, and reliability; however, they are generally limited by the required sophisticated instruments, tedious sample preparation, and highly trained/skilled technicians. Thus, they are not suitable for routine screening of suspected contaminants in many samples [5]. A gold nanoparticle (AuNP)-based immunochromatographic assay (ICA) has been widely used for on-site detection of various mycotoxins because of its simplicity, rapidity (less than 10 min), and naked-eye detection [10]. However, it is commonly limited by poor sensitivity. The enzyme-linked immunosorbent assay (ELISA) has relatively high sensitivity, because it involves the usage of horseradish peroxidase (HRP)-catalyzed tetramethylbenzidine for signal amplification. However, the insufficient signal intensity of the resulting products, with an extinction coefficient as low as 5.9 × 10^4^ M^−1^ cm^−1^, limited the detection sensitivity of a conventional ELISA [11].

Compared with a conventional ELISA, the plasmonic enzyme-linked immunoassay (pELISA), based on the localized surface plasmon resonance (LSPR) of AuNPs as signal output, shows a higher sensitivity because the extinction coefficient of AuNPs is 4–5 order higher than that of the traditional chromophoric group [11,12,13,14,15,16,17,18,19]. Distinct LSPR change can be induced by slight changes of the compositions, shapes, sizes or aggregation states of AuNPs, and thus promotes various AuNPs-based colorimetric assays for detection of antigens, nucleic acids, mycotoxins and microorganisms. Among all these strategies, the controlled growth kinetics strategy was widely used due to its high detection sensitivity and good robustness [15,20,21,22]. AuNPs with a size of 30–40 nm exhibit a bright purple color, whereas the small-sized AuNP solution (~5 nm) is colorless at the same molar concentration because of its lower molar extinction coefficient. When the solution contains gold seeds (5 nm AuNPs), HAuCl_4_·3H_2_O, and reductant, such as hydrogen peroxide (H_2_O_2_), the Au^3+^ is prone to reduction into the Au atom on the surface of gold seeds. The small-sized AuNPs are grown into large-sized AuNPs, thereby resulting in a dramatic color change from colorless to purple, which is easy to be observed by the naked eye.

In this study, a novel pELISA based on the controlled growth kinetics of AuNPs was developed for naked-eye detection of FB_1_ in food samples. The combination of the ultrahigh sensitivity of pELISA and the vivid color change generated from AuNPs growth contributed to the high sensitivity of the proposed method for FB_1_ visual inspection, with a cut-off limit of 1.25 ng/mL. This method also showed a good linear range of 0.31 ng/mL to 10 ng/mL for FB1 quantitative detection, with a half maximal inhibitory concentration (IC_50_) of 1.86 ng/mL. The accuracy, robustness, and reliability of the proposed pELISA were evaluated by analyzing FB_1_-spiked agricultural products and further comparing the results with the conventional ELISA method. The results demonstrated that the proposed pELISA has a great potential for sensitive detection of FB_1_ contamination by the naked eye or a plate reader, especially in resource-constrained regions.

## 2. Results and Discussion

### 2.1. Principle of the Proposed pELISA Method

**Figure 1:**
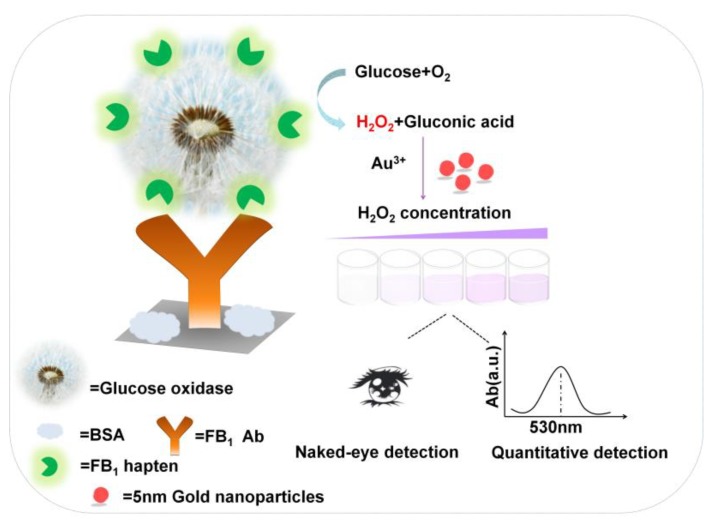
Schematic of Glucose oxidase (GOx) induced plasmonic enzyme-linked immunoassay (pELISA) for the detection of fumonisin B_1_ (FB_1_) in maize.

The principle of the proposed method is shown in Figure 1. Glucose oxidase (GOx) was used as an alternative to HRP as the carrier of competing antigen. In the absence of FB_1_ in the solution, GOx-FB_1_ conjugates (GOx@FB_1_) were captured by the antibodies that were immobilized on the wells and catalyzed glucose to generate H_2_O_2_. Au^3+^ was reduced to Au atom on the surface of 5 nm gold seeds (which was originally diluted to be colorless with a concentration of 5 nM), resulting in an increase in the size of the AuNPs and a remarkable color change from colorless to purple red. Conversely, less GOx was captured on the plate wells, resulting in lower growth of AuNPs and color change of the solution.

### 2.2. Feasibility of GOx Regulated AuNPs Growth 

**Figure 2:**
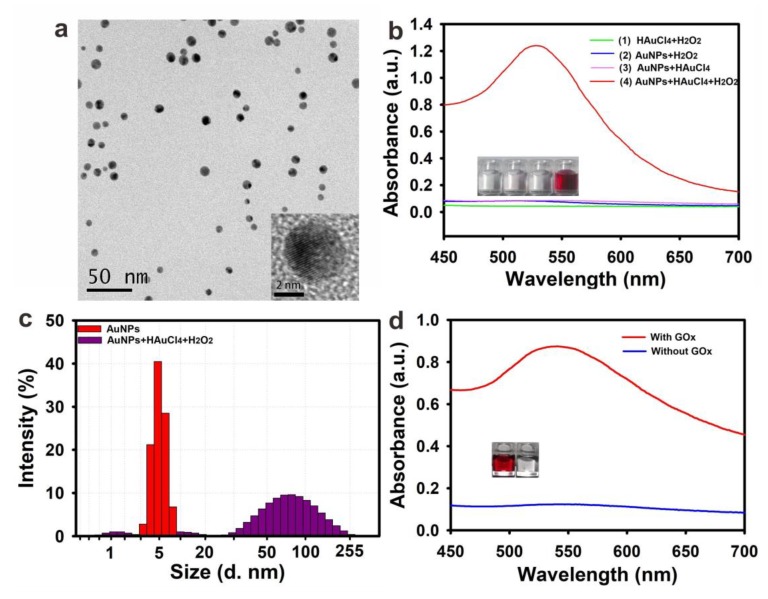
(**a**) TEM image of 5 nm gold nanoparticles (AuNPs). (**b**) Generation of colored solution for the detection with naked eyes. Each group’s solutions were recorded with their UV-vis spectrum and the corresponding digital photos. (**c**) The hydration dynamic diameter of 5 nm AuNPs. (**d**) Verification of the reducing of 5 nm AuNPs in the presence of GOx; UV-vis spectrum and the corresponding color.

As is reported, the extinction coefficient of small-sized AuNPs, such as 5 nm, is much lower than those of larger-sized AuNPs [13]. Thus, the diluted small-sized AuNPs at a relatively low concentration were colorless. These kinds of small-sized AuNPs are easy to grow into larger-sized AuNPs with a red or purple red color in the presence of H_2_O_2_ and HAuCl_4_ to produce a vivid color contrast. In this study, the small-sized AuNPs were synthesized according to a previously described method [23]. The TEM image in Figure 2a shows that the as-prepared gold seeds displayed a narrow size distribution with an average diameter of 5.0 nm ± 0.5 nm (*n* = 50). The UV-visible (UV-vis) spectra (Figure S1a) revealed that the gold seed solution exhibited a maximum surface plasma resonance (SPR) peak at 520 nm. The Dynamic light scattering (DLS) analysis (Figure S1b) indicated that the average hydrodynamic diameter of gold seeds was 5.5 nm ± 0.3 nm (*n* = 3) with a polydispersity index (PDI) at 0.191, indicating an excellent monodispersity of the synthesized gold seed solution. Furthermore, no aggregation and flocculation were observed after the storage of the as-prepared gold seed solution at 4 °C for six months.

To verify the feasibility of the H_2_O_2_-mediated of AuNPs growth, four tracers, including (1) HAuCl_4_ + H_2_O_2_, (2) gold seed solution + H_2_O_2_, (3) gold seed solution + HAuCl_4_, and (4) gold seed solution + HAuCl_4_ + H_2_O_2_, were performed. To obtain a high signal-to-noise ratio, the optimal H_2_O_2_ and HAuCl_4_ concentrations were 100 μM and 0.4 mM, respectively, because the excess contents of H_2_O_2_ and HAuCl_4_ are prone to directly reduce HAuCl_4_ into the Au atom in the absence of gold seeds, which could generate a color background that could interfere with the naked-eye observation [13]. The gold seed solution was diluted to colorless (5 nM). The results in the inset of the Figure 1b showed that the color of the solution in the group (4) turned into purple red, indicating that the HAuCl_4_ can be reduced into the Au atom in the addition of gold seeds and H_2_O_2_ in generating large-sized AuNPs. The red-shift SPR peak at 530 nm of the purple red solution further demonstrated the growth of AuNPs. The TEM image in Figure S2 showed that the shape of AuNPs changed from a regular sphere to irregular morphology with an increased size of 45 nm ± 13 nm. The DLS analysis (Figure 2c) showed that the average hydrodynamic diameter of AuNPs also increased to 67 nm ± 3 nm, whereas the PDI value increased to 0.375, indicating a non-uniform size distribution of grown AuNPs. The irregular size and morphology of grown AuNPs were ascribed to the reduced Au atoms that were clustered and deposited on the surfaces of the gold seeds when the H_2_O_2_ concentration is at a low level. This result is similar with the previous report [13]. Furthermore, we also investigated the impact of GOx on the growth of AuNPs. With the catalytic oxidation of glucose to generate H_2_O_2_ using GOx, the gold solution observably turned into purple red with an obvious SPR peak at 530 nm (Figure 2d). This result demonstrated that the H_2_O_2_ generated from the oxidation of GOx to glucose can also induce the gold seed growth mode and accordingly paves the way for further developed GOx-mediated pELISA.

### 2.3. Optimization of the Parameters of pELISA

In this study, the GOx was used as an alternative for HRP in the development of a direct competitive pELISA. The competing antigen was prepared by coupling the carboxyl group of FB_1_ molecule with the amino group of GOx. The UV–visible spectra of FB_1_, GOx, and GOx@FB_1_ conjugates are shown in Figure S3. The results displayed that the FB_1_ molecule have no characteristic absorption peak in the range of 200 nm to 350 nm. The results showed that, compared with the FB_1_ molecule, an obvious characteristic absorption peak in the range of 280 nm was observed on GOx@FB_1_ conjugates, and the peak was with a slight red shift with further comparison of GOx, indicating the successful conjugation of the FB_1_ molecule and GOx. To further verify whether the FB_1_ molecule was coupled with the GOx, the GOx@FB_1_ conjugate was bound to an anti-FB_1_ mAbs pre-coated onto the plate well. The glucose substrate was added after washing the unbound GOx@FB_1_ conjugate, and the bioactivity of GOx was evaluated by determining the concentration of the generated H_2_O_2_. The results in the inset of Figure S3 show that the GOx was bound on the plate well by the FB_1_ and mAbs interaction, further showing that the GOx@FB_1_ conjugate was successfully prepared for subsequent analysis. 

In the directly competitive pELISA, the concentrations of the coating antibody (anti-FB_1_ ascitic fluids) and the competitive antigen were two key parameters that influenced the detection sensitivity [24]. The optimal concentrations of the coating antibody and the competitive antigen were obtained through a checkerboard titration method. The increase in the concentrations of the coating antibody and GOx@FB_1_ resulted to the gradual change from colorless to purple red of the solution color in the plate wells (Figure S4) and the sharp rise in the optical density at 530 nm (OD_530_) (Table S1). When the concentrations of the coating antibody and GOx@FB_1_ were 2.00 μg/mL and 1.56 μg/mL, respectively, the solution color in the plate well appeared as clear purple red with an OD_530_ value at 0.15, which was easily identified by the naked eye. The lower concentrations of coating antibody and GOx@FB_1_ were not enough to produce significant color contrast, thereby reducing the signal-to-noise ratio. Thus, the optimal concentrations of anti-FB_1_ mAbs and GOx@FB_1_ were set at 2.00 μg/mL and 1.56 μg/mL, respectively. 

**Figure 3:**
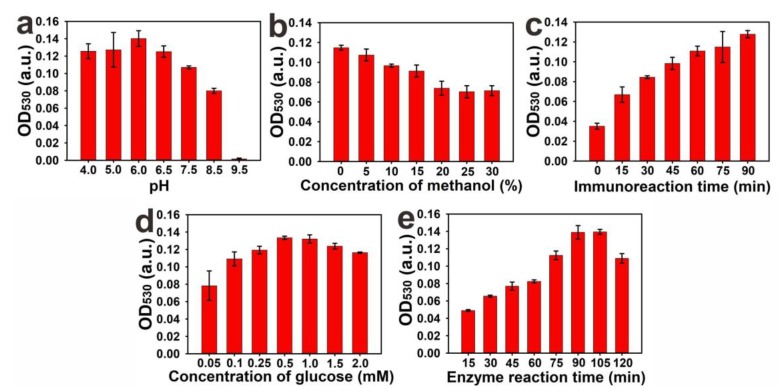
Parameters optimized in pELISA. (**a**) pH values of sample extraction solvent (4.0–9.5); (**b**) extraction solvent; methanol (*V/V*, 0%~30%); (**c**) immunoreaction time (0–90 min); (**d**) Glucose concentration (0.05–2.0 M) as substrate of enzymatic reaction for H_2_O_2_ generation and (**e**) enzyme reaction time (15–120 min). The error bars indicate standard deviation of three measurements. (*n* = 3).

Moreover, previous studies demonstrated that pH value, methanol content, and immunoreaction time could significantly influence the immunoreactions between the antigen and the antibody, thereby reducing the detection sensitivity of the immunoassay [24,25]. Thus, the determination of the effects of the pH on the immunoassay was performed by adjusting the pH within the range of 4.0 to 9.5. As shown in Figure 3a, the OD_530_ values showed a relatively high level at a relatively low pH, indicating that the weak acid condition (pH = 4.0–6.0) may be conducive to the interaction of the FB_1_ molecule and mAbs. Increasing the pH from 6.0 to 8.5 resulted to the gradual decrease of the OD_530_ value from 0.141 to 0.075. At pH 9.5, the OD_530_ value sharply decreased to 0.002. To ensure an efficient immunological response, pH 6.0 was considered as the optimal pH condition for the subsequent experiments. The extraction solution containing a certain concentration of methanol can effectively reduce the matrix interference of the protein or water-soluble components from maize samples. However, antibody-antigen interaction can be influenced by a high content of methanol [26]. Thus, we investigated the effects of different methanol concentrations ranging from 0% to 30% on the immunoassay. As depicted in Figure 3b, the OD_530_ values significantly decreased with the increase in methanol content from 0% to 30%, indicating that the methanol concentration has a great impact on the interaction of FB_1_ and mAbs. Considering the high sensitivity of the proposed method, the actual extraction solution was suggested to be diluted with pH 6.0 PBS (0.01 M) to a final methanol concentration of 5% for further analysis. The results of the effects of immunoreaction time between mAbs and FB_1_@GOx on the immunoassay are shown in Figure 3c. The results indicate that the OD_530_ value gradually increased and did not reached a plateau when the reaction time was extended to 90 min. However, the solution color exhibited an obvious purple red after 60 min. Therefore, 60 min of immunoreaction time was sufficient to ensure a high ratio of signal to noise for the naked-eye observation. In addition, the concentration of glucose and the time of GOx catalyzed-glucose oxidation could also impact the H_2_O_2_ production, further affecting the sensitivity of pELISA. Figure 3d indicates that the OD_530_ values reached the maximum when the glucose concentration was 0.5 M. An excessive glucose concentration could result in the occurrence of substrate inhibition effect, which in turn would decrease the catalytic efficiency of GOx and lower the OD_530_ value. Additionally, the catalysis reaction time between FB_1_@GOx and glucose substrate were investigated. Figure 3e shows that the OD_530_ value increased greatly by prolonging the reaction time. It then reached a plateau when the catalysis time was 90 min. Collectively, the optimized experimental conditions were described as follows: the 0.01 M PBS with pH of 6.0 was used for mAbs and antigen immunoreactions; the real sample extraction containing 60% methanol should be further diluted to a final concentration of 5%; the immunoreaction time between mAbs and antigen was set at 60 min; 0.5 M glucose was used as the enzyme substrate; and reaction time of GOx catalysis catalyzed-glucose oxidation was set at 90 min. 

### 2.4. Analytical Performance of pELISA for the Sensitive Detection of FB_1_

**Figure 4:**
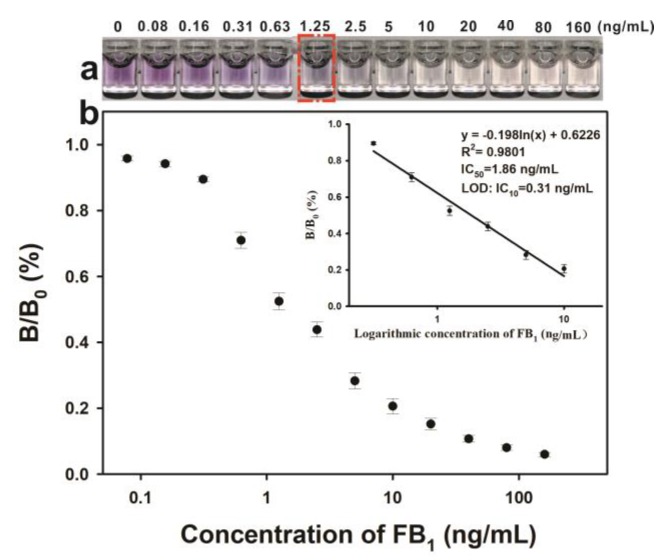
(**a**) Digital photos taken of the immunoassays for detection of FB_1_ at the concentrations of 0–160 ng/mL (the one with red border was with the concentration of FB_1_ 1.25 ng/mL, the visible cut-off limit by naked eyes); (**b**) Inhibition curve for FB_1_ as obtained by plotting the normalized signal B/B_0_ × 100% against the logarithm of FB_1_ concentration.

Under the optimal experimental conditions, the competitive inhibition curve of the pELISA was developed. Figure 4a shows that the color of the solution obviously changed from purple red to colorless by increasing the FB_1_ concentration from 0 ng/mL to 1.25 ng/mL. The tonality from purple red to colorless was easily distinguishable by the naked eye. Therefore, 1.25 ng/mL of FB_1_ was defined as the visible cut-off limit by the naked eye. Quantitative analysis of the proposed pELISA method was performed based on the FB_1_ calibration curve. The calibration curve was constructed by plotting the B/B_0_ against different FB_1_ concentrations (0 ng/mL, 0.08 ng/mL, 0.16 ng/mL, 0.31 ng/mL, 0.63 ng/mL, 1.25 ng/mL, 2.5 ng/mL, 5 ng/mL, 10 ng/mL, 20 ng/mL, 40 ng/mL, 80 ng/mL, and 160 ng/mL), where B and B_0_ represented the OD_530_ values of sample with and without FB_1_, respectively. Figure 4b shows that the developed pELISA exhibited a good linear range of 0.31 ng/mL to 10 ng/mL with a reliable correlation coefficient (R^2^) at 0.9801. The regression equation could be represented by *y* = −0.198 ln(*x*) + 0.6226, where *y* is B/B_0_ and *x* is the FB_1_ concentration. The error bars were based on quadruplicate measurements. The IC_50_ of the obtained pELISA was achieved at 1.86 ng/mL (Figure S5), which is approximately 13-fold lower than that of HRP-based conventional ELISA (IC_50_ = 25 ng/mL). The detection limit (LOD) of the proposed pELISA was calculated as 0.31 ng/mL based on the concentration of IC_10_ value. This value is further comparable to other established immunoassays for FB_1_ detection (Table S2). The proposed pELISA in this work displayed quite excellent sensitivity in FB_1_ detection. 

### 2.5. Selectivity of the Proposed Sensing System

**Figure 5:**
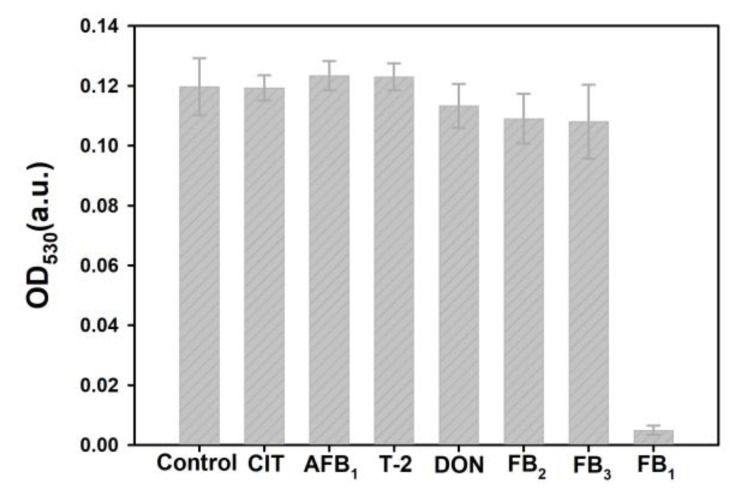
The selectivity of pELISA. FB_1_ spiked concentration was 0.1 μg/mL; citinin (CIT), aflatoxin B_1_ (AFB_1_), T-2, deoxyivalenol (DON), FB_2_, and FB_3_ spiked concentrations were 1 μg/mL; negative control test was performed by adding the PBS solution containing 5% (*v/v*) methanol. The error bars indicate standard deviation of three measurements. (*n* = 3).

The selectivity of our developed pELISA was performed by analyzing FB_1_ (0.1 μg/mL) and other common mycotoxins, including CIT, AFB_1_, T-2, DON, FB_2_, and FB_3_ (1 μg/mL). Meanwhile, a negative control was conducted using PB buffer (pH 6.0, 0.01 M, containing 5% methanol). Figure 5 shows that the decreased OD_530_ value was only observed in the presence of FB_1_, and negligible changes (*p* > 0.05) were observed in other common mycotoxins and the control experiment. These results showed that the developed pELISA exhibits high selectivity for FB_1_ determination because of the specific recognition of FB_1_ and anti-FB_1_ mAbs.

### 2.6. Validation of pELISA on Maize Samples

**Figure 6:**
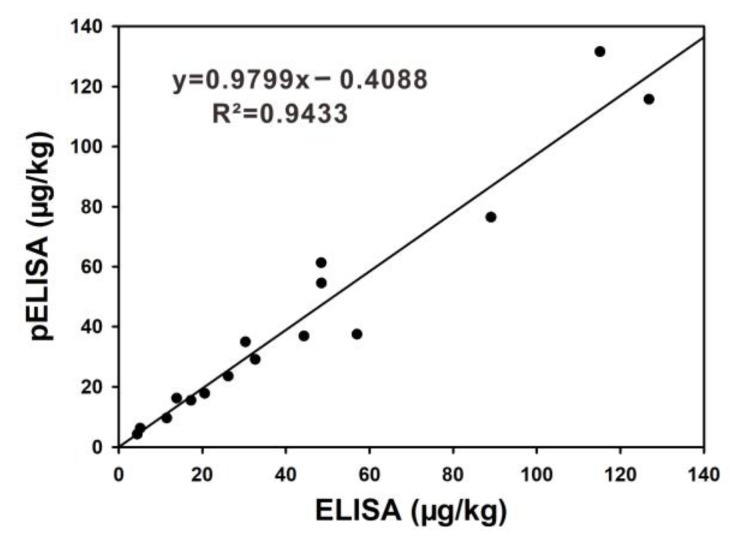
Methodology comparison between the pELISA and horseradish peroxidase- (HRP)-ELISA.

The accuracy and precision of the proposed pELISA for FB_1_ quantitative detection was evaluated through the addition and recovery analysis. The real maize substrates were spiked by different concentrations of FB_1_ (0.08 mg/kg, 0.15 mg/kg, 0.30 mg/kg, 1.20 mg/kg, and 2.40 mg/kg) and were analyzed through the proposed method. The results in Table 1 show that the average recoveries of the five spiked samples varied from 88.33% to 116.67% with a coefficient of variation (CV) ranging from 2.54% to 13.20%. These results indicate an acceptable accuracy and precision in the detection of FB_1_. Later, a comparison analysis was carried out through conventional ELISA to estimate the reliability of our proposed method. In the comparison analysis, 16 artificially contaminated FB_1_ maize samples were simultaneously determined by the proposed pELISA and conventional ELISA, results see Table S3. Figure 6 shows an excellent correlation between these two approaches (R^2^ = 0.9433), indicating that the proposed pELISA could be applied for reliable determination of FB_1_ in real maize products.

## 3. Conclusions

We successfully developed a pELISA for sensitive naked-eye detection of FB_1_ using GOx as HRP substitute and colloidal gold solution as a color signal output. GOx, which is low-cost and high catalytic efficiency, and catalyze the oxidization of glucose into H_2_O_2_ and gluconic acid. The resultant H_2_O_2_ then reduces Au^3+^ to Au^0^ on the surface of 5 nm AuNPs to induce an obvious solution color change from colorless to purple red. Under the optimum conditions, the visual cut-off value of our developed method for naked-eye detection of FB_1_ was 1.25 ng/mL, and the IC_50_ was as low as 1.86 ng/mL, which is about 13-fold lower than that of conventional ELISA. In addition, the proposed method displayed an acceptable precision and accuracy, as well as an excellent correlation with conventional HRP-based ELISA for FB_1_ detection. In brief, the developed pELISA showed a great potential for the sensitive naked-eye detection of mycotoxins or other small molecular chemicals in food safety monitoring. Furthermore, its high-throughput screening detection ability and naked eyes easy readout without advanced detection equipment is considerably suitable for point-of-care diagnostics in resource-constrained regions.

## 4. Materials and Methods 

### 4.1. Regents

Sodium borohydride (NaBH_4_), HAuCl_4_·3H_2_O, trisodium citrate, maleic anhydride, protein G, bovine serum albumin (BSA), GOx, and glucose, FB_1_, fumonisin B_2_ (FB_2_), fumonisin B_3_ (FB_3_), citinin (CIT), deoxyivalenol (DON), aflatoxin B_1_ (AFB_1_) and Trichothecenes 2 toxins (T-2), 1-(3-Dimethylaminopropyl)-3-ethylcarbodiimide hydrochloride (EDC), 3, 3’, 5, 5’-Tetramethylbenzidine liquid system and H_2_O_2_ were purchased from Sigma-Aldrich Chemical (St. Louis, MO, USA). An H_2_O_2_ quantitative assay kit (water-compatible) was purchased from Sangon Biotech (Shanghai, China) (www.sangon.com). Anti-FB_1_ monoclonal antibody ascitic fluids (anti-FB_1_ mAb 6.5 mg/mL) were provided by Prof. Xu research group (Nanchang University, China). 96-well microplates (high binding, white) were obtained from Costar Inc. (Cambridge, MA, USA). Other reagents were of analytical grade were purchased from Sinopharm Chemical Corp (Shanghai, China). Aqueous solutions for the immunoassay experiments were prepared using Millipore water (Elix-3þ Milli-QA, Millipore, France).

### 4.2. Apparatus

All the absorption spectrums were recorded by Thermo Fisher 1510-03690 (Vantaa, Finland). The size of gold seeds was measured through transmission electron microscopy (JEM-2100HR, JEOL, Tokyo, Japan), and the average hydrodynamic diameter and monodispersity of the gold seeds were characterized by (Malvern Instruments Ltd. U.K, Malvern, UK).

### 4.3. The Synthesis of Gold Seeds

Gold seeds were synthesized according to a previous report with slight modifications [23]. In brief, 10 mL of trisodium citrate solution (0.5 mM) containing 0.5 mM of HAuCl_4_ solution was put into a washed conical flask while stirring constantly at room temperature for 2 min. Then, 0.6 mL NaBH_4_ solution (0.1 M, dissolved in ice water) was added to the solution while stirring vigorously for another 2 min. The reaction was immediately terminated by chilling the resulting solution in a mixture of ice water for 45 min. The resultant gold seed solution was stored at 4 °C until further use. 

### 4.4. Preparation of FB_1_-Labeled GOx

FB_1_-labeled GOx conjugates (GOx@FB_1_) were prepared according to a carbodiimide method [27]. In brief, 135 μL of FB_1_ (1 mg/mL) solution was added to 1 mL GOx solution (1.5 mg/mL, and mole ratio of FB_1_:GOx is 20:1). Added with 50 μL freshly prepared EDC solution (7.2 mg/mL), the mixture was under constant stirring at room temperature for 2 h and then dialyzed in PBS (0.01 M, pH 6.2) at 4 °C for 72 h. The obtained GOx@FB_1_ complex was stored in 50% (w/v) glycerol solution at −20 °C until further use.

### 4.5. GOx Mediated Direct Competitive pELISA

The proposed pELISA method was conducted based on the H_2_O_2_ generated from the reaction between GOx and glucose, wherein the amount of H_2_O_2_ regulated the growth of gold seeds along with different color responses. In brief, the 96 well microplates were coated with 100 μL protein-G solution (20 μg/mL) at 4 °C overnight. The plates were washed thrice with phosphate buffer containing 0.05% Tween-20 (PBST, pH 7.4, 0.01 M) to remove unbound protein-G and blocked with 10 mg/mL of BSA solution at 37 °C for 1 h. After washing with PBST for three times, 100 μL of anti-FB_1_ ascitic fluids (2 μg/mL) was added into each plate well. The plates were then incubated at 37 °C for 1 h and were washed again for three times. About 50 μL of sample solution and 50 μL of GOx@FB_1_ (1.56 μg/mL) were added into each well at 37 °C for 1 h. After performing the same washing procedure, we added 100 μL of glucose (0.5 M) solution and incubated the plates at 37 °C for 1 h. Then, 100 μL of growth solution containing 0.4 mM HAuCl_4_ and 5 nM gold seeds was added into each well, and then the plates were kept for another 1 h at ambient temperature. The absorbance of each well at 530 nm was recorded using a microplate reader. The detailed operation of conventional HRP-based ELISA was described in the Supplementary Materials. 

Notably, seven parameters including the concentrations of the coating antibody (anti-FB1 ascitic fluids) and the competitive antigen, the pH value and methanol concentration of sample solution, the concentration of glucose, the immunoreaction time and enzymatic reaction time were optimized. The concentrations of the coating antibody and the competitive antigen were optimized first under preset conditions, where sample solution was set as 0.01 M PBS (pH = 7, 0% methanol), the concentration of glucose was 1 M in ultra-pure water, the immunoreaction time and enzymatic reaction time were both 1 h. The other parameters were optimized successively under the above conditions with corrections made according to previous results. 

### 4.6. Sample Preparation

The maize samples, which were confirmed to be free of FB_1_ through the LC-MS/MS method, were collected from the supermarket in Jiangxi and Shandong Provinces in China. To evaluate the accuracy and precision of the proposed method, different amounts of FB_1_ stock solution (200 µg/mL) were mixed with 1 g of well ground maize powder to make the FB_1_ final concentrations at 0.08 mg/kg, 0.15 mg/kg, 0.30 mg/kg, 1.20 mg/kg, and 2.40 mg/kg. In addition, 16 maize samples were artificially contaminated with FB_1_ concentrations in the range of 5.0 μg/kg–150 μg/kg for the reliability evaluation of the proposed method. The FB_1_-spiked maize samples were extracted according to a previous method with some modifications [28]. Briefly, 5 mL of PB buffer (0.01 M, pH 6.0, 10 mM NaCl, and 60% methanol) was added into 1 g of ground maize powder while vigorously shaking on a plate shaker for 20 min. The mixture was centrifuged at 6000 *g* for 10 min. The supernatant was further diluted to 12-fold for pELISA (reaching 5% methanol) and conventional ELISA analysis.

## Figures and Tables

**Table 1:** Accuracy of the plasmonic enzyme-linked immunoassay (pELISA) in fumonisin B_1_ (FB_1_)-spiked samples.

Spiked FB_1_ (mg/kg)	Average ^a^	Recovery (%)	Standard Deviation	CV (%)
2.40	2.28	95.04	2.41	2.54
1.20	1.06	88.33	10.16	11.85
0.30	0.35	116.67	14.89	13.20
0.15	0.17	113.33	8.15	6.89
0.08	0.08	104.00	2.81	3.32

^a^ Mean value of 5 replicates at each spiked concentration.

## Supplementary Materials

The following are available online at https://www.mdpi.com/2072-6651/11/6/323/s1, Figure S1: Characterization of the sythesised 5 nm AuNPs with Uv-vis spectrum (a) and hydrodynamic diameter (b), Figure S2: The morphology of 5 nm AuNPs before (a) and after grown (b), Figure S3: The UV-vis spectrum of glucose oxidase (GOx), fumonisin B_1_ (FB_1_) and GOx@FB_1_, indicating that GOx has been successfully immobilized onto the FB_1_, Figure S4: The chessboard titration experiment and the photo taken is in accordance with the figures displayed in Table S1, Figure S5: The calibration curve of the conventional HRP based ELISA, Table S1: The selection for the working conditions of GOx@FB_1_ and anti-FB_1_ ascitic fluids based plasmonic enzyme-linked immunoassay (pELISA) using the checkerboard method, Table S2: Comparison of this work with some established immunoassays for FB_1_ detection, Table S3: Comparision of ELISA with pELISA analysis of fumonisin B_1_ in 16 artificially contaminated maize samples.
