# Supplementary material for: A Gold Growth-Based Plasmonic ELISA for the Sensitive Detection of Fumonisin B1 in Maize
Source: Toxins (Basel). 2019 Jun 5;11(6):323. doi: 10.3390/toxins11060323 (PMC6628417; doi:10.3390/toxins11060323)
Supplement: Supplementary file 1 [file toxins-11-00323-s001.pdf]

Supplementary Materials: A Gold Growth-Based Plasmonic ELISA for The Sensitive Detection of Fumonisin B1 in Maize

Shengnan Zhan, Lingyan Zheng, Yaofeng Zhou, Kesheng Wu, Hong Duan, Xiaolin Huang and Yonghua Xiong

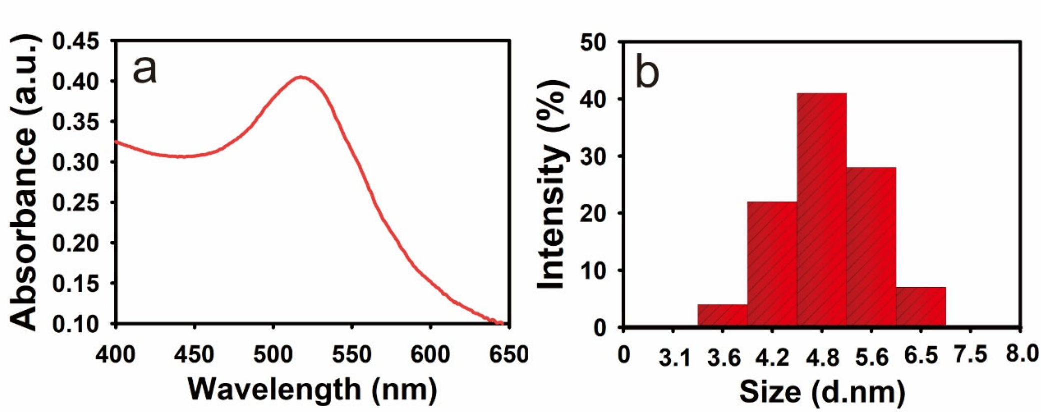

Figure S1. Characterization of the sythesised 5 nm AuNPs with Uv-vis spectrum (**a**) and hydrodynamic diameter (**b**).

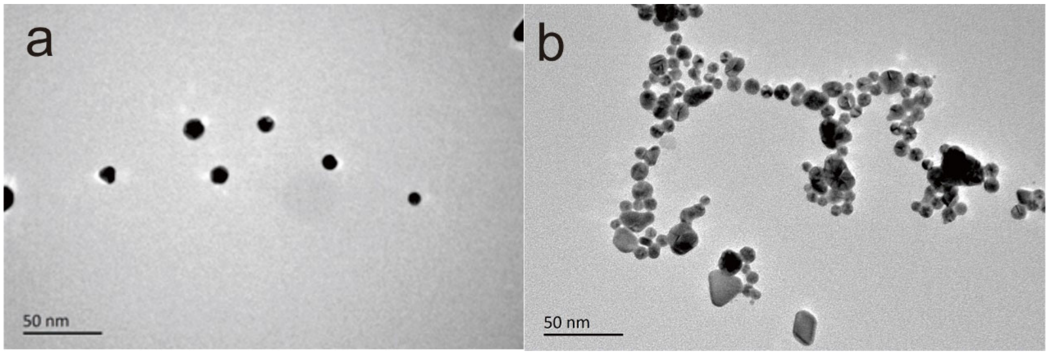

Figure S2. The morphology of 5 nm AuNPs before (**a**) and after grown (**b**).

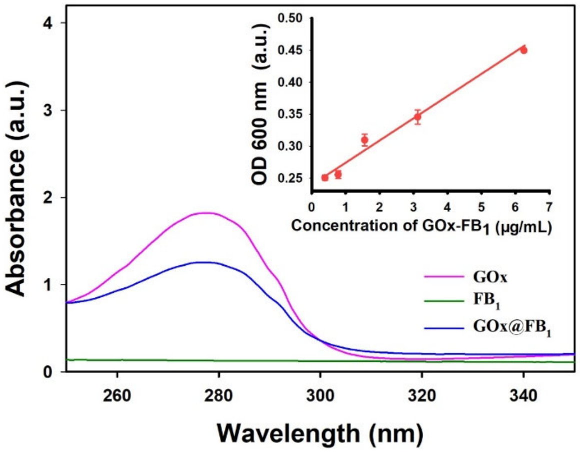

FigureS3. The UV-vis spectrum of glucose oxidase (GOx), fumonisin B_1 (FB_1) and GOx@FB_1, indicating that GOx has been successfully immobilized onto the FB_1.

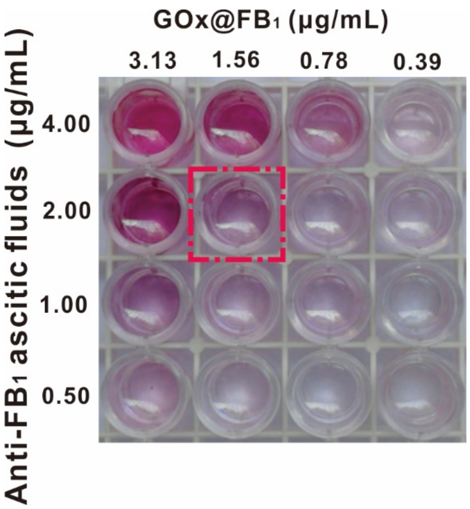

Figure S4. The chessboard titration experiment and the photo taken is in accordance with the figures displayed in Table S1.

1. The Verification of GOx Conjugated FB_1

The insert graph is the characterization of the H_2O_2 produced by the reaction between glucose oxidase (GOx) and glucose after the enzyme-linked immunosorbant assay using a H_2O_2 quantitative assay kit (water-compatible) method, details were as follow: 96-well microplate which was coated with protein G (20 μg/mL, diluted with 0.01 M PBS, pH 8.6) at at 4 °C overnight, then the plate was washed with PBST (0.01 M PBS, with 0.05% tween-20, pH 7.4) thrice, anti-FB_1 mAbs (2.00 μg/mL, 100 μL per well) was incubated at 37 °C, 1 h. With the same procedure, then plate was blocked with 1 mg/mL BSA solution for 2 h at 37 °C. Different concentrations of GOx-FB_1 was added with 2-fold diluted (0, 0.39, 0.78, 1.56, 3.13, 6.25 μg/mL) at 37 °C, 1 h. After three times washing with PBST, 1 M of glucose solution (100 μL per well) was added and incubated at 37 °C for another 1 h, then 10 μL of each reaction solution was moved to the work solution (100 μL) from the H_2O_2 quantitative assay kit (water-compatible). After incubating 2 h at 37 °C, the absorbance value at 600 nm was recorded by a microplate reader (DNM-9602, PERLONG, Beijing, China).

2. The Conventional HRP Based ELISA

To compare the sensitivity with the proposed pELISA, we conducted the horse radish peroxidase (HRP) conventional ELISA based on the same molecular ratio of HRP to FB_1 compared to GOx to FB_1. In brief, the conventional ELISA was performed as follow: The 96-well microplate was incubated with 100 μL per well of protein G (20 μg/mL, diluted with 0.01 M PBS, pH 8.6) at 4 °C overnight, the unbounded protein was washed with PBST (0.01 M PBS, with 0.05% tween-20, pH 7.4) thrice, then 300 μL of blocking buffer (1 mg/mL BSA solution) was added to block the redundant sites for a period of 1 h at 37 °C, after that, the plate was washed three times with washing buffer, and 100 μL of anti-FB_1 ascetic fluids (2.03 μg/mL) were added into each well and incubated at 37 °C for 1 h, subsequently, the plate was washed three times with washing buffer, and 50 μL of FB_1-HRP and 50 μL of sample solution was added to each well, followed by incubating at 37 °C for 60 min. The same washing procedure was performed to remove the redundant element, then 100 μL TMB solution (50 μL solution A and 50 μL solution B mixed immediately) was added into each well, then after 15 min of incubation at 37 °C, 50 μL stop buffer (sulfuric acid, 2 mg/mL) was added to stop the reaction, then the absorbance of the plate was recorded by a microplate reader (DNM-9602, PERLONG, Beijing, China).

The parameters of conventional ELISA were optimized by a checkerboard titration method. The optimized concentrations of anti-FB_1 ascitic fluids and FB_1-HRP were 5 μg/mL and 2.03 μg/mL, respectively. Under the optimized, the IC_{50} value of the conventional ELISA was 25 ng/mL, which was about 13-fold higher than that of the proposed plasmonic-ELISA method (Figure S2).

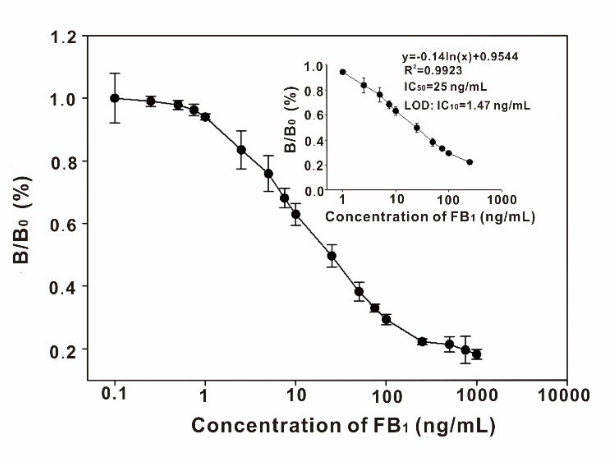

Figure S5. The calibration curve of conventional ELISA. Vertical bars indicate the standard deviation (n = 3).

Table S1. The selection for the working conditions of GOx@FB_1 and anti-FB_1 ascitic fluids based plasmonic enzyme-linked immunoassay (pELISA) using the checkerboard method.

**Ascitic Fluids Concentration (μg/mL)**	**GOx@FB_1（μg/mL）**			
	**3.13**	**1.56**	**0.78**	**0.39**
4.00	0.29	0.19	0.12	0.08
2.00	0.21	0.15*	0.09	0.08
1.00	0.14	0.11	0.09	0.07
0.50	0.09	0.08	0.06	0.05

* The data in red, whose corresponding parameters of anti-FB_1 ascitic fluids and GOx@FB_1 ware the final selected ones.

Table S2. Comparison of this work with some established immunoassays for FB_1 detection

Method	Material Used	LOD	LOD by Naked Eyes	References
ELISA	Magnetic beads	0.24 ng/mL	/	[1]
Fluoresence-based ICA	Fluorescent microspheres	0.12 ng/mL	/	[2]
Fluorescence polarization immunoassay	Fluorescein	157.4 μg/kg	/	[3]
Electrochemical	Single-walled carbon nanotubes/chitosan	2 pg/mL	/	[4]
Microarray	Mimotope, fluorescein	11.1 ng/mL	/	[5]
Chemiluminescence	Gold-coated magnetic nanoparticles	0.027 ng/mL	/	[6]
Electrochemiluminescence	$Ru(bpy)_3^{2+}$-doped silica nanoparticles	0.35 μg/kg	/	[7]
Immunochromatographic strip	Gold nanoparticles	25 ng/mL	/	[8]
Paper sensor	Gold nanoparticle	0.53–1.05μg/kg	5–25 μg/kg	[9]
Plasmonic ELISA	Gold nanoparticles	0.31 ng/mL	1.25 ng/mL	This study

Table S3. Comparision of ELISA with pELISA analysis of fumonisin B1 in 16 artificially contaminated maize samples.

Sample	Contaminated Concentration (μg/kg)	Detected ELISA (μg/kg)	Detected pELISA (μg/kg)
1	5	5.14	6.19
2	10	11.49	9.59
3	15	13.85	16.19
4	15	17.28	15.48
5	15	20.55	17.78
6	20	26.19	23.54
7	20	30.32	34.94
8	25	32.66	29.10
9	25	44.30	36.90
10	32	48.42	61.27
11	35	48.50	54.50
12	50	57.01	37.46
13	60	89.08	76.45
14	100	115.19	131.56
15	150	126.89	115.70

References

1. Wang, Y.-K.; Wang, Y.-C.; Wang, H.-a.; Ji, W.-H.; Sun, J.-H.; Yan, Y.-X. An immunomagnetic-bead-based enzyme-linked immunosorbent assay for sensitive quantification of fumonisin B_1. *Food Control.* **2014**, *40*, 41–45.
2. Wang, Z.; Li, H.; Li, C.; Yu, Q.; Shen, J.; De Saeger, S. Development and application of a quantitative fluorescence-based immunochromatographic assay for fumonisin b1 in maize. *J. Agric. Food Chem.* **2014**, *62*, 6294–6298.
3. Li, C.; Mi, T.; Oliveri Conti, G.; Yu, Q.; Wen, K.; Shen, J.; Ferrante, M.; Wang, Z. Development of a screening fluorescence polarization immunoassay for the simultaneous detection of fumonisins B_1 and B_2 in maize. *J. Agric. Food Chem.* **2015**, *63*, 4940–4946.
4. Yang, X.; Zhou, X.; Zhang, X.; Qing, Y.; Luo, M.; Liu, X.; Li, C.; Li, Y.; Xia, H.; Qiu, J. A highly sensitive electrochemical immunosensor for fumonisin B_1 detection in corn using single-walled carbon nanotubes/chitosan. *Electroanalysis* **2015**, *27*, 2679–2687.

5. Peltomaa, R.; Benito-Peña, E.; Barderas, R.; Sauer, U.; González Andrade, M.; Moreno-Bondi, M.C. Microarray-based immunoassay with synthetic mimotopes for the detection of fumonisin B_1. *Anal. Chem.* **2017**, *89*, 6216–6223.
6. Jie, M.; Yu, S.; Yu, F.; Liu, L.; He, L.; Li, Y.; Zhang, H.; Qu, L.; Harrington, P.d.B.; Wu, Y. An ultrasensitive chemiluminescence immunoassay for fumonisin B_1 detection in cereals based on gold-coated magnetic nanoparticles. *J. Sci. Food Agric.* **2018,** *98(9)*, 3384–3390.
7. Zhang, W.; Xiong, H.; Chen, M.; Zhang, X.; Wang, S. Surface-enhanced molecularly imprinted electrochemiluminescence sensor based on Ru@ SiO_2 for ultrasensitive detection of fumonisin B_1. *Biosens. Bioelectron.* **2017**, *96*, 55–61.
8. Hao, K.; Suryoprabowo, S.; Hong, T.; Song, S.; Liu, L.; Zheng, Q.; Kuang, H. Immunochromatographic strip for ultrasensitive detection of fumonisin B_1. *Food Agric. Immunol.* **2018**, 1–12.
9. Kong, D.; Liu, L.; Song, S.; Suryoprabowo, S.; Li, A.; Kuang, H.; Wang, L.; Xu, C. A gold nanoparticle-based semi-quantitative and quantitative ultrasensitive paper sensor for the detection of twenty mycotoxins. *Nanoscale* **2016**, *8*, 5245–5253.

© 2019 by the authors. Submitted for possible open access publication under the terms and conditions of the Creative Commons Attribution (CC BY) license (http://creativecommons.org/licenses/by/4.0/).
